# Age-specific risk of malignancy in pediatric thyroid cytology: Reframing ROM based on pre-test probability

**DOI:** 10.1210/clinem/dgag073

**Published:** 2026-02-25

**Authors:** Sule Canberk, Zubair W Baloch, Amber Isaza, Mya Bojarsky, Elena Vigliar, Anna Maria Carillo, Filippo Dello Iacovo, Claudio Bellevicine, Giancarlo Troncone, Mariana Simplício, Helena Barroca, Serra Zeynep Akkoyunlu, Mehmet Güven Günver, Elisabete Rodrigues, João Capela, Vivian L Weiss, Huiying Wang, Ozlem Aydin, Eleni Thodou, Hasan Gücer, Fernando Schmitt, Andrew J Bauer

**Affiliations:** Department of Pathology, Faculty of Medicine, RISE-Health, University of Porto (FMUP), 4200-319 Porto, Portugal; Department of Pathology and Laboratory Medicine, Children's Hospital of Philadelphia, University of Pennsylvania, Philadelphia, PA 19104, USA; Division of Endocrinology and Diabetes, The Thyroid Center, Children's Hospital of Philadelphia, Philadelphia, PA 19104, USA; Division of Endocrinology and Diabetes, The Thyroid Center, Children's Hospital of Philadelphia, Philadelphia, PA 19104, USA; Department of Public Health, University of Naples Federico II, 80131 Naples, Italy; Department of Public Health, University of Naples Federico II, 80131 Naples, Italy; Department of Public Health, University of Naples Federico II, 80131 Naples, Italy; Department of Public Health, University of Naples Federico II, 80131 Naples, Italy; Department of Public Health, University of Naples Federico II, 80131 Naples, Italy; Department of Pathology, Faculty of Medicine, RISE-Health, University of Porto (FMUP), 4200-319 Porto, Portugal; Department of Pathology, Unidade Local de Saúde de São João, 4200-319 Porto, Portugal; Department of Biostatistics, Istanbul Medical Faculty, Basic Medical Sciences, 34093 Istanbul, Türkiye; Department of Biostatistics, Istanbul Medical Faculty, Basic Medical Sciences, 34093 Istanbul, Türkiye; Department of Endocrinology, ULS São João and Faculty of Medicine, University of Porto, 4200-319 Porto, Portugal; Endocrine and Neck Surgery Division, Department of Surgery, ULS São João and Faculty of Medicine, University of Porto, 4200-319 Porto, Portugal; Department of Pathology, Microbiology, and Immunology, Vanderbilt University Medical Center, Nashville, TN 37232, USA; Department of Pathology, Microbiology, and Immunology, Vanderbilt University Medical Center, Nashville, TN 37232, USA; Department of Pathology, School of Medicine, Acibadem Mehmet Ali Aydinlar University, 34752 Istanbul, Turkey; Department of Pathology, Faculty of Medicine, School of Health Sciences, University of Thessaly, Larissa 41500, Greece; Private Cytology Laboratory, Volos, Thessaly 38221, Greece; Department of Pathology, School of Medicine, Recep Tayyip Erdoğan University, 53100 Rize, Turkiye; Department of Pathology, Faculty of Medicine, RISE-Health, University of Porto (FMUP), 4200-319 Porto, Portugal; Division of Endocrinology and Diabetes, The Thyroid Center, Children's Hospital of Philadelphia, Philadelphia, PA 19104, USA; Department of Paediatrics, Perelman School of Medicine, Children's Hospital of Philadelphia, Philadelphia, PA 19104, USA

**Keywords:** pediatric thyroid nodules, thyroid fine-needle aspiration, thyroid cytopathology, Bethesda system (TBSRTC), likelihood ratios, risk of malignancy

## Abstract

**Objective:**

Pediatric thyroid nodules are uncommon but have a higher malignancy rate than adult nodules. Existing Bethesda risk-of-malignancy (ROM) estimates are not age-stratified and are affected by verification bias. We aimed to generate age-specific ROM, likelihood ratios (LRs), and post-test malignancy probabilities for pediatric and young adult thyroid cytology.

**Methods:**

We analysed 2728 thyroid fine-needle aspirations (FNAs) from patients aged 0-25 years across multiple tertiary centers (2000-2023). All cases were classified or reclassified using the 2023 Bethesda System and grouped into four age bands (0-8, 9-14, 15-18, and 19-25 years). We calculated lower-bound ROM (ROM overall; assuming nonoperated FNAs were benign) and surgery-only upper-bound ROM (ROM surgery; 991 of 2728 cases with histology). Age-specific pretest probabilities and Bethesda-category LRs were used to compute post-test malignancy probabilities with Bayes’ theorem.

**Results:**

Among operated nodules, malignancy prevalence decreased with age (84.2% at 0-8 years to 64.6% at 19-25 years). Verification bias was substantial: malignancy prevalence was 24.6% (671/2728) under the lower-bound assumption but 67.7% (671/991) among surgical cases. Upper-bound ROM values were 2-10 times higher than lower-bound values across categories. Benign cytology showed low LRs (0.07-0.15), reducing post-test malignancy probability to 0-25%. Indeterminate categories showed age-related variation; for example, a follicular neoplasm diagnosis carried a ∼71% post-test risk in a 9-year-old versus ∼49% in a 24-year-old. AUS subtyping (nuclear vs other atypia) did not consistently separate ROM.

**Conclusion:**

Age substantially modifies both pretest and post-test malignancy probabilities in pediatric thyroid cytology. An age-stratified LR framework helps quantify verification bias and provides individualized risk estimates to guide decisions about surgery, molecular testing, and follow-up.

Thyroid nodules in children and adolescents are uncommon (0.5-2% in most series) but are enriched for malignancy compared with those in adult, prompting more intensive evaluation and follow-up (1, 2). In parallel, the incidence of pediatric differentiated thyroid cancer has increased over recent decades (3). Despite frequent presentation with regional nodal disease (4) and occasional distant metastases (5), outcomes remain excellent, showing important biological differences from adult thyroid cancer (3).

The 2023 third edition of the Bethesda System for Reporting Thyroid Cytopathology (TBSRTC) (6) incorporated pediatric-specific risk-of-malignancy (ROM) estimates, highlighting that indeterminate cytology categories carry higher malignancy risks in children than in adults. These differences influence clinical decision-making, including surgical planning, extent of thyroidectomy, and the potential role of molecular testing (2, 5). However, TBSRTC still applies a single set of pediatric ROM values across a broad age range (typically all patients younger than 19 years), despite increasing evidence that prepubertal children and adolescents differ in clinical behavior and molecular drivers. Large registry data indicate that younger children present with more invasive disease than older adolescents, and molecular studies show an age gradient from fusion-driven tumors in younger children toward increasing prevalence of point mutations (including BRAF V600E) with advancing age (4, 7-12). Treating all pediatric patients as a single cohort may therefore obscure clinically meaningful heterogeneity.

A further limitation is methodological. ROM is a post-test probability that depends on the pretest probability (baseline prevalence) in the population being evaluated. Published ROM values are susceptible to selection and verification bias because histological confirmation is disproportionately obtained in clinically high-suspicion nodules (13). As discussed by Baloch and colleagues (13), these biases and context-dependence limit the portability and patient-level interpretability of ROM tables across practices and across patient subgroups. LRs offer a complementary approach: they summarize diagnostic performance in a prevalence-independent way and can be used with Bayes’ theorem to generate patient-specific post-test probabilities, while allowing sequential updating as additional information becomes available (eg, ultrasound risk pattern or molecular results) (14, 15). Despite these advantages, LR-based frameworks have not been systematically implemented for age-stratified pediatric thyroid cytology.

We therefore conducted a multi-institutional study to generate age-stratified ROM estimates and corresponding LRs for thyroid FNA cytology across four developmental periods (0-8, 9-14, 15-18, and 19-25 years). We also quantified the impact of verification bias by reporting lower-bound (all cases) and upper-bound (surgical cases only) estimates and provided results under both exclusive and inclusive histological definitions for low-risk borderline tumors, to support clinically transparent and patient-specific risk assessment.

## Materials and methods

### Study design and participants

This was a retrospective, multi-institutional cohort study of pediatric and young adult patients (age ≤25 years at time of FNA) who underwent thyroid fine-needle aspiration at seven tertiary referral centers in Italy, the USA, Portugal, Türkiye, and Greece between 2000 and 2023. The study was approved by the institutional review boards of participating institutions, with waiver of informed consent granted due to its retrospective design. The Committees for the Protection of Human Subjects at the Children's Hospital of Philadelphia reviewed the PEDIMAP protocol and determined it to be exempt (CHOP IRB #23-021615; 45 CFR 46.104(d)(4)(iii); April 29, 2024). A waiver of HIPAA authorization was granted (45 CFR 164.512(i)(2)(ii)). Data were analysed in de-identified form.

Eligible FNAs included those with an interpretable cytology diagnosis that could be assigned (prospectively or retrospectively) to a 2023 TBSRTC category and had outcome data available from surgical histopathology or clinical follow-up. FNAs performed before local adoption of the Bethesda System were originally reported using institution-specific terminology; for this study, we retrospectively mapped those diagnoses (pre-2009 cases: *n* = 5) to 2023 TBSRTC categories using the original report wording and diagnostic criteria.

FNAs were excluded if they could not be reliably mapped to 2023 TBSRTC, if outcome data were missing, or if patient age at FNA was unavailable. Of 3498 extracted records, 617 were excluded for missing age (yielding 2881 age-eligible FNAs), and another 153 were excluded for missing, invalid, or nonmappable cytology, resulting in a final analytic cohort of 2728 FNAs. Of these, 991 (36.3%) had histological confirmation and 1737 (63.7%) were managed nonsurgically. Per-center FNA totals are summarized in Table 1.

**Table 1:** Per-center numbers and Bethesda category distribution

Center	Total (n)	Histology available (n)	Surgery available (n)	N malignant (Excl) (n)	Surgery rate (%)	Malignancy prevalence among histology available cases (%) (Excl)	I (%)	II (%)	III (%)	IV (%)	V (%)	VI (%)
C1	1689	130	131	73	7.7	55.7	8.3	65.4	12.0	0.8	3.6	9.9
C2	420	376	376	312	89.5	83.0	1.4	21.2	10.5	11.9	10.0	45.0
C3	172	170	171	91	98.8	53.2	1.2	31.4	22.1	4.1	6.4	34.9
C4	135	132	134	66	97.8	49.3	11.1	28.1	7.4	20.0	5.9	27.4
C5	119	118	118	79	99.2	66.9	1.7	18.5	17.6	14.3	17.6	30.3
C6	105	27	27	22	25.7	81.5	27.6	46.7	5.7	1.9	1.9	16.2
C7	88	34	34	28	38.6	82.4	0.0	53.4	9.1	5.7	4.5	27.3

C1 = University of Naples Federico II, Naples, Italy, C2 = Children's Hospital of Philadelphia, USA, C3 = Vanderbilt University Medical Center, USA, C4 = Faculty of Medicine, University of Porto, Portugal, C5 = Acibadem University, Istanbul, Türkiye, C6 = Recep Tayyip Erdoğan University, Türkiye, C7 = Private Cytology Laboratory, Volos, Thessaly, Greece/Faculty of Medicine, University of Thessaly, Larissa, Greece. N total = all FNAs evaluated at the center. N surgery = cases that underwent surgery. N with histology = operated cases with a histologic diagnosis available. N malignant (excl) = Number of histology-proven malignant tumors, *excluding* low-risk/borderline neoplasms, among operated cases with histology available. Surgery rate % = (N surgery / N total) × 100. Malignancy prevalence, surgical % (exclusive) = (N malignant [exclusive] / N with histology) × 100. Columns I-VI = distribution of TBSRTC categories (ND, B, AUS, FN, SFM, M, respectively) among all FNAs at that center (row percentages); values in I-VI sum to ∼100% per center (minor differences from rounding). NA = not estimable. Exclusive endpoint (Excl): counts only histology-proven malignant tumors and explicitly excludes low-risk/borderline neoplasms. Inclusive endpoint (Incl): counts malignant + low-risk/borderline neoplasms as events.

### Procedures

Cytology diagnoses were prospectively assigned (Bethesda-era FNAs) or retrospectively mapped from original report wording (pre-Bethesda-era FNAs) to 2023 TBSRTC categories: I, nondiagnostic; II, benign; III, atypia of undetermined significance (AUS); IV, follicular neoplasm (FN); V, suspicious for malignancy (SFM); and VI, malignant.

Patients were stratified into four developmental age groups based on clinical relevance and prior literature (4, 7-12):

1. **0-8 years** (Early Childhood).
2. **9-14 years** (Preadolescence/Early Adolescence).
3. **15-18 years** (Late Adolescence).
4. **19-25 years** (Young Adulthood).

### Outcomes

Surgical histopathology served as the reference standard for determining malignancy in resected nodules. Nonoperated nodules were managed with clinical follow-up according to local practice.

Because classification and clinical handling of borderline thyroid tumors vary, we coded histological outcomes using two parallel definitions: (1) an exclusive definition, in which borderline tumors [noninvasive follicular thyroid neoplasm with papillary-like nuclear features (NIFTP), well-differentiated tumor of uncertain malignant potential (WDT-UMP), and hyalinizing trabecular tumour] were classified as benign; and (2) an inclusive definition, in which these borderline tumors were classified as malignant.

### Statistical analysis

For each age-band × Bethesda-category stratum, we estimated risk of malignancy (ROM) using two approaches to bracket verification bias: lower-bound ROM (ROM overall), calculated as malignant cases divided by all FNAs in the stratum, assuming all nonoperated nodules were benign; and upper-bound ROM (ROM surgery), calculated as malignant cases divided by surgically treated FNAs in the same stratum. Both ROM estimates were computed under exclusive and inclusive outcome definitions (four ROM estimates per stratum). Exact 95% confidence intervals (CIs) for proportions were calculated using the Clopper-Pearson method.

Within each age group, pretest probability was defined as the prevalence of malignancy among surgically treated nodules (exclusive and inclusive); pretest = (Total malignant cases in age group with surgery)/(Total cases in age group with surgery). Among surgically treated nodules in each age band, we calculated LRs for each Bethesda category as the probability of that category in histologically malignant nodules divided by the probability of the same category in histologically benign nodules. In counts, LR = (*n* malignant in category/total malignant) ÷ (*n* benign in category/total benign). Post-test odds were calculated as pretest odds × LR and converted to post-test probabilities using Bayes’ theorem (Post-test probability = Post-test odds/(1 + Post-test odds)); in this dataset, these post-test probabilities correspond numerically to ROM surgery for the same age-band × category. We calculated 95% confidence intervals for LRs on the log scale; when no benign cases occurred in a category, the LR was infinite and the upper confidence bound was reported as infinity.

We compared ROM and LRs across age groups using χ^2^ tests or Fisher's exact tests (expected counts <5) and assessed ordered trends across age bands using the Cochran-Armitage test for trend (two-sided *P* < .05). We also summarized per-center case counts and Bethesda distributions and performed a leave-one-center-out sensitivity analysis of age-specific pretest prevalence. Analyzes were done in Python (version 3.9), with code independently verified by two analysts.

### Role of the funding source

This study was supported by The Children's Hospital of Philadelphia Frontier Program, which provided internal institutional support that contributed to development of the Child & Adolescent Thyroid Consortium (CATC) framework. The funder had no role in study design; data collection, analysis, or interpretation; writing of the report; or the decision to submit for publication.

## Results

### Cohort characteristics

We analysed 2728 thyroid fine-needle aspirations (FNAs) from patients aged 0-25 years, collected across seven tertiary referral centers between 2000 and 2023. Most patients were female (2234/2728; 81.9%); 491 (18.0%) were male, and sex was missing for three (0.1%). FNAs were most frequently performed in young adults aged 19-25 years (1628/2728; 59.7%), followed by ages 15-18 years (631; 23.1%), 9-14 years (432; 15.8%), and 0-8 years (37; 1.4%).

Overall, 991 FNAs (36.3%) had surgical histology, while 1737 (63.7%) were managed nonsurgically. Surgical verification varied by TBSRTC category: lowest in benign (13.8%; 193/1403) and nondiagnostic (15.9%; 31/195) FNAs, highest in follicular neoplasm (91.7%; 111/121), and intermediate in AUS (45.0%; 148/329), SFM (69.1%; 103/149), and malignant categories (76.3%; 405/531). Center-level case volumes, surgical verification rates, malignancy yield, and Bethesda distributions are summarized in Table 1. Among pediatric nondiagnostic FNAs (ages 0-18) 15 of 60 (25.0%) proceeded to surgery. Nonoperated nondiagnostic nodules with available composition data were cystic in 17 of 42 cases (40.5%), whereas operated nondiagnostic nodules were more often cystic (7/12; 58.3%) and larger (median 25 mm vs 14 mm).

Using the exclusive outcome definition, the lower-bound malignancy proportion was 24.6% (671/2728), assuming all nonoperated nodules were benign, and 67.7% (671/991) among surgically resected nodules. Using the inclusive definition, malignancy proportions were 25.4% (693/2728) overall and 69.9% (693/991) among resected nodules (Table 2).

**Table 2:** Age-stratified cytologic diagnostic categories with ROM, likelihood ratios, and updated probabilities (ages 0-25 years)

AG-G	TBSRTC	N (All)	N (Surg.)	ROM% (Overall, Excl) (95% CI)	ROM% (Overall, Incl) (95% CI)	ROM% (Surg., Excl) (95% CI)	ROM% (Surg., Incl) (95% CI)	Pre-test P (excl)	Pre-test P (incl)	LR (Excl) (95% CI)	LR (Incl), (95% CI)	Post-test P (Excl)	Post-test P (Incl)
0-8	ND	3	0	0.0% (0.0-70.8%)	0.0% (0.0-70.8%)	NA	NA	84.2%	84.2%	NA	NA	NA	NA
0-8	Benign	16	2	0.0% (0.0-20.6%)	0.0% (0.0-20.6%)	0.0% (0.0-84.2%)	0.0% (0.0-84.2%)	84.2%	84.2%	0.00 (0.00-1.00)	0.00 (0.00-1.00)	0.0%	0.0%
0-8	AUS	4	3	50.0% (6.8-93.2%)	50.0% (6.8-93.2%)	66.7% (9.4-99.2%)	66.7% (9.4-99.2%)	84.2%	84.2%	0.38 (0.02-22.12)	0.38 (0.02-22.12)	66.7%	66.7%
0-8	SFM	2	2	100.0% (15.8-100.0%)	100.0% (15.8-100.0%)	100.0% (15.8-100.0%)	100.0% (15.8-100.0%)	84.2%	84.2%	∞ (0.04-∞)	∞ (0.04-∞)	100.0%	100.0%
0-8	Malignant	12	12	100.0% (73.5-100.0%)	100.0% (73.5-100.0%)	100.0% (73.5-100.0%)	100.0% (73.5-100.0%)	84.2%	84.2%	∞ (0.52-∞)	∞ (0.52-∞)	100.0%	100.0%
**0-8**	**Total**	**37**	**19**	**43.2% (27.1-60.5%)**	**43.2% (27.1-60.5%)**	**84.2% (60.4-96.6%)**	**84.2% (60.4-96.6%)**	**84.2%**	**84.2%**	**NA**	**NA**	**NA**	**NA**
9-14	ND	16	3	6.2% (0.2-30.2%)	6.2% (0.2-30.2%)	33.3% (0.8-90.6%)	33.3% (0.8-90.6%)	72.8%	74.5%	0.19 (0.00-3.58)	0.17 (0.00-3.29)	33.3%	33.3%
9-14	Benign	191	43	3.7% (1.5-7.4%)	4.7% (2.2-8.8%)	16.3% (6.8-30.7%)	20.9% (10.0-36.0%)	72.8%	74.5%	0.07 (0.03-0.17)	0.09 (0.04-0.19)	16.3%	20.9%
9-14	AUS	48	33	29.2% (17.0-44.1%)	31.2% (18.7-46.3%)	42.4% (25.5-60.8%)	45.5% (28.1-63.6%)	72.8%	74.5%	0.27 (0.13-0.58)	0.29 (0.13-0.60)	42.4%	45.5%
9-14	FN	29	28	69.0% (49.2-84.7%)	69.0% (49.2-84.7%)	71.4% (51.3-86.8%)	71.4% (51.3-86.8%)	72.8%	74.5%	0.93 (0.39-2.45)	0.86 (0.36-2.25)	71.4%	71.4%
9-14	SFM	32	30	93.8% (79.2-99.2%)	93.8% (79.2-99.2%)	100.0% (88.4-100.0%)	100.0% (88.4-100.0%)	72.8%	74.5%	∞ (2.85-∞)	∞ (2.62-∞)	100.0%	100.0%
9-14	Malignant	116	106	90.5% (83.7-95.2%)	91.4% (84.7-95.8%)	99.1% (94.9-100.0%)	100.0% (96.6-100.0%)	72.8%	74.5%	39.15 (6.88-1560.98)	∞ (9.67-∞)	99.1%	100.0%
**9-14**	**Total**	**432**	**243**	**41.0% (36.3-45.8%)**	**41.9% (37.2-46.7%)**	**72.8% (66.8-78.3%)**	**74.5% (68.5-79.8%)**	**72.8%**	**74.5%**	**NA**	**NA**	**NA**	**NA**
15-18	ND	41	12	4.9% (0.6-16.5%)	4.9% (0.6-16.5%)	16.7% (2.1-48.4%)	16.7% (2.1-48.4%)	66.8%	68.4%	0.10 (0.01-0.47)	0.09 (0.01-0.43)	16.7%	16.7%
15-18	Benign	290	64	5.2% (2.9-8.4%)	5.5% (3.2-8.8%)	23.4% (13.8-35.7%)	25.0% (15.0-37.4%)	66.8%	68.4%	0.15 (0.08-0.28)	0.15 (0.08-0.28)	23.4%	25.0%
15-18	AUS	85	51	25.9% (17.0-36.5%)	28.2% (19.0-39.0%)	43.1% (29.3-57.8%)	47.1% (32.9-61.5%)	66.8%	68.4%	0.38 (0.21-0.68)	0.41 (0.23-0.74)	43.1%	47.1%
15-18	FN	40	38	60.0% (43.3-75.1%)	60.0% (43.3-75.1%)	63.2% (46.0-78.2%)	63.2% (46.0-78.2%)	66.8%	68.4%	0.85 (0.42-1.78)	0.79 (0.39-1.66)	63.2%	63.2%
15-18	SFM	30	24	76.7% (57.7-90.1%)	80.0% (61.4-92.3%)	95.8% (78.9-99.9%)	100.0% (85.8-100.0%)	66.8%	68.4%	11.45 (1.86-471.48)	∞ (2.79-∞)	95.8%	100.0%
15-18	Malignant	145	127	86.2% (79.5-91.4%)	86.9% (80.3-91.9%)	98.4% (94.4-99.8%)	99.2% (95.7-100.0%)	66.8%	68.4%	31.10 (8.43-259.65)	58.33 (10.28-2322.10)	98.4%	99.2%
**15-18**	**Total**	**631**	**316**	**33.4% (29.8-37.3%)**	**34.2% (30.5-38.1%)**	**66.8% (61.3-71.9%)**	**68.4% (62.9-73.4%)**	**66.8%**	**68.4%**	**NA**	**NA**	**NA**	**NA**
19-25	ND	135	16	3.7% (1.2-8.4%)	3.7% (1.2-8.4%)	31.2% (11.0-58.7%)	31.2% (11.0-58.7%)	64.6%	67.8%	0.25 (0.07-0.78)	0.22 (0.06-0.67)	31.2%	31.2%
19-25	Benign	906	84	1.4% (0.8-2.4%)	1.8% (1.0-2.9%)	15.5% (8.5-25.0%)	19.0% (11.3-29.1%)	64.6%	67.8%	0.10 (0.05-0.18)	0.11 (0.06-0.19)	15.5%	19.0%
19-25	AUS	192	61	13.5% (9.0-19.2%)	16.7% (11.7-22.7%)	42.6% (30.0-55.9%)	52.5% (39.3-65.4%)	64.6%	67.8%	0.41 (0.23-0.69)	0.52 (0.31-0.90)	42.6%	52.5%
19-25	FN	52	45	42.3% (28.7-56.8%)	48.1% (34.0-62.4%)	48.9% (33.7-64.2%)	55.6% (40.0-70.4%)	64.6%	67.8%	0.52 (0.28-0.98)	0.59 (0.32-1.13)	48.9%	55.6%
19-25	SFM	85	47	50.6% (39.5-61.6%)	51.8% (40.7-62.7%)	91.5% (79.6-97.6%)	93.6% (82.5-98.7%)	64.6%	67.8%	5.88 (2.14-22.55)	6.97 (2.23-35.07)	91.5%	93.6%
19-25	Malignant	258	160	61.2% (55.0-67.2%)	61.2% (55.0-67.2%)	98.8% (95.6-99.8%)	98.8% (95.6-99.8%)	64.6%	67.8%	43.20 (11.76-359.82)	37.53 (10.22-312.56)	98.8%	98.8%
**19-25**	**Total**	**1628**	**413**	**16.4% (14.6-18.3%)**	**17.2% (15.4-19.1%)**	**64.6% (59.8-69.3%)**	**67.8% (63.1-72.3%)**	**64.6%**	**67.8%**	**NA**	**NA**	**NA**	**NA**

AG-G = age group (0-8, 9-14, 15-18, 19-25 years). TBSRTC (2023): ND (I), Benign (II), AUS (III; incl. FLUS), FN (IV; incl. SFN), SFM (V), Malignant (VI). N(All) = all FNAs in the age-band × category cell. N (Surg.) = cases in that cell with surgery and histology available. ROM% (Overall, Excl/Incl) = *lower-bound* risk of malignancy using all FNAs (nonoperated counted as benign); Exclusive endpoint (Excl): counts only histology-proven malignant tumors and explicitly excludes low-risk/borderline neoplasms.; Inclusive endpoint (Incl): counts malignant + low-risk/borderline neoplasms as events ((in this cohort: NIFTP and tumors of uncertain malignant potential [FT-UMP/WDT-UMP]; hyalinizing trabecular tumor was prespecified but not observed).). ROM% (Surg., Excl/Incl) = *upper-bound* risk of malignancy among surgically treated cases with histology. Pretest P (excl/incl) = age-band malignancy prevalence among surgically treated nodules under the respective definition (same value for all categories within an age band). LR (Excl/Incl) = age-band likelihood ratio for each TBSRTC category (see Materials and Methods for calculation). Post-test P (Excl/Incl) = probability of malignancy after applying the LR to the age-band pretest prevalence; in this table, it coincides with the corresponding surgery-only ROM for that cell. 95% CIs: exact (Clopper–Pearson) for ROM; standard log-scale for LR. NA = not estimable; totals may not sum to 100% due to rounding.

### Age-specific pretest probability of malignancy

**Figure 1:**
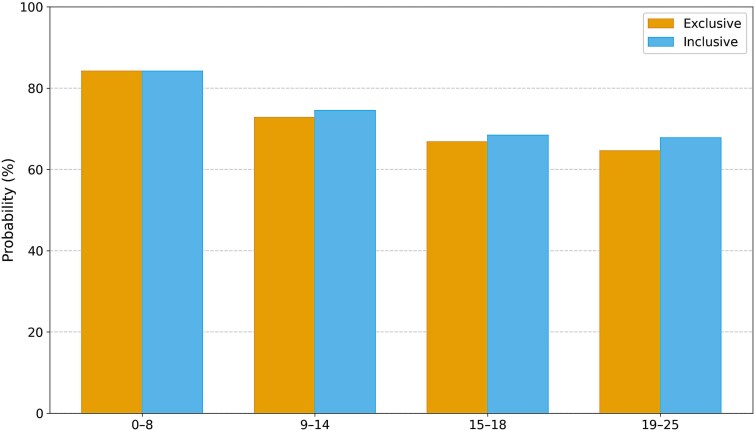
Pretest probability of malignancy by age group. Pretest probability is defined as the malignancy prevalence among surgically treated nodules within each age band (0-8, 9-14, 15-18, and 19-25 years). Bars show estimates under the exclusive histology definition (borderline/low-risk neoplasms counted as benign) and the inclusive definition (borderline/low-risk neoplasms counted as malignant). Values correspond to Table 2; LOOC sensitivity results are shown in Table 3.

Pretest probability (malignancy prevalence among surgically treated nodules) decreased with age (Fig. 1; Table 2). Under the exclusive definition, pretest probability was 84.2% for ages 0-8 years, 72.8% for ages 9-14 years, 66.8% for ages 15-18 years, and 64.6% for ages 19-25 years (Table 2). Under the inclusive definition, corresponding values were 84.2%, 74.5%, 68.4%, and 67.8% (Table 2). The absolute difference between the youngest and oldest age bands was 19.6% points under the exclusive definition (84.2% vs 64.6%; Table 2).

In leave-one-center-out sensitivity analyses (LOOC), the stepwise age-gradient persisted in 6 of 7 runs, and ages 0-8 years remained the highest-risk stratum in every run. Pretest estimates for midadolescents varied when the center with the highest histology verification rate was excluded (Table 3).

**Table 3:** Leave-one-center-out pretest prevalence by age (%, exclusive definition)

Scenario	0-8	9-14	15-18	19-25
Overall (all centers)	84.2	72.8	66.8	64.6
Exclude C2	85.7	52.2	47.8	63.1
Exclude C4	83.3	74.1	70.4	67.8
Exclude C3	86.7	80.9	69.3	65.2
Exclude C1	84.2	73.3	69.7	66.0
Exclude C7	84.2	72.7	67.0	63.0
Exclude C5	82.4	72.6	66.8	64.5
Exclude C6	84.2	72.7	66.7	63.7

“Overall (all centers)” reports the age-band pretest prevalence using all seven centers. The rows “Exclude C1”, “Exclude C2”, “Exclude C3”, “Exclude C4”, “Exclude C5”, “Exclude C6”, “Exclude C7” recompute the same quantity after removing that specific center. Pretest prevalence (exclusive) within each age band (0-8, 9-14, 15-18, 19-25 years) is (# histology-proven malignant tumors, excluding low-risk/borderline neoplasms) − (# with histology among surgically treated nodules), pooled across the remaining centers using raw counts (not center-level averages). Values are percentages (one decimal). Center codes: C1 = University of Naples Federico II, Naples, Italy; C2 = Children's Hospital of Philadelphia, USA; C3 = Vanderbilt University Medical Center, USA; C4 = Faculty of Medicine, University of Porto, Portugal; C5 = Acibadem University, Istanbul, Türkiye; C6 = Recep Tayyip Erdoğan University, Türkiye; C7 = Private Cytology Laboratory, Volos, Thessaly, Greece/Faculty of Medicine, University of Thessaly, Larissa, Greece.

Abbreviation: LOOC, leave-one-center-out sensitivity analysis.

### Age-stratified risk of malignancy and Bayesian likelihood ratios

**Figure 2:**
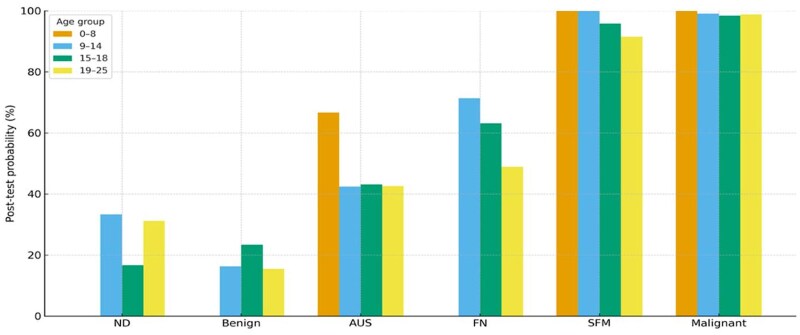
Post-test probability of malignancy by Bethesda category and age (exclusive definition). Bars show the post-test probability of malignancy for each age band × TBSRTC category under the exclusive definition. Post-test probabilities are obtained by applying the age-specific pretest probability and the age-specific likelihood ratio (Table 2) via Bayes’ theorem; in this dataset, these values coincide numerically with the corresponding surgery-only ROM for each stratum. Category counts (N overall; N with surgery) are provided in Table 4. Abbreviations: ND (I) = nondiagnostic; Benign (II); AUS (III) = atypia of undetermined significance; FN (IV) = follicular neoplasm; SFM (V) = suspicious for malignancy; Malignant (VI).

Age-stratified ROM estimates (lower-bound ROM overall and ROM surgery-(surgery-only), LRs, and the corresponding post-test probabilities for each Bethesda category are shown in Table 2 (with case counts by age-band × Bethesda in Table 4). Post-test probabilities by TBSRTC category and age are summarized in Fig. 2 (exclusive definition) inclusive-definition results were similar and are shown numerically in Table 2.

**Table 4:** Case counts by age band and Bethesda category with number undergoing surgery

AG-G	TBSRTC	N (All)	N (Surg.)
**0-8**	**ND**	3	0
**0-8**	**Benign**	16	2
**0-8**	**AUS**	4	3
**0-8**	**FN**	0	0
**0-8**	**SFM**	2	2
**0-8**	**Malignant**	12	12
**0-8 subtotal**		**37**	**19**
**9-14**	**ND**	16	3
**9-14**	**Benign**	191	43
**9-14**	**AUS**	48	33
**9-14**	**FN**	29	28
**9-14**	**SFM**	32	30
**9-14**	**Malignant**	116	106
**9-14 subtotal**		**432**	**243**
**15-18**	**ND**	41	12
**15-18**	**Benign**	290	64
**15-18**	**AUS**	85	51
**15-18**	**FN**	40	38
**15-18**	**SFM**	30	24
**15-18**	**Malignant**	145	127
**15-18 subtotal**		**631**	**316**
**19-25**	**ND**	135	16
**19-25**	**Benign**	906	84
**19-25**	**AUS**	192	61
**19-25**	**FN**	52	45
**19-25**	**SFM**	85	47
**19-25**	**Malignant**	258	160
**19-25 subtotal**		**1628**	**413**
**Grand total**		**2728**	**991**

AG-G = age group (0-8, 9-14, 15-18, 19-25 years). TBSRTC (2023): ND (I), Benign (II), AUS (III; incl. FLUS), FN (IV; incl. SFN), SFM (V), Malignant (VI). N (All) = all FNAs in the age-band × category cell. N (Surg.) = cases in that cell that underwent surgery. *Subtotals are sums across categories within each age band*.

### High-risk cytology categories (TBSRTC V and VI)

Across age bands, categories V (SFM) and VI (malignant) were associated with very high post-test probabilities of malignancy in the surgically treated subset (ROM surgery). For category VI, ROM surgery ranged from 98.4% to 100.0% (exclusive definition) and from 98.8% to 100.0% (inclusive definition); corresponding LRs were very large (LR excl 31.10 to ∞; LR incl 37.53 to ∞). For category V, ROM surgery ranged from 91.5% to 100.0% (exclusive) and from 93.6% to 100.0% (inclusive); LRs ranged from 5.88 to ∞ (exclusive) and from 6.97 to ∞ (inclusive) (Table 2; Fig. 2).

### Low-risk cytology categories (TBSRTC I and II)

For category II (benign), ROM overall ranged from 0.0% to 5.2% (exclusive) and from 0.0% to 5.5% (inclusive), whereas ROM surgery ranged from 0.0% to 23.4% (exclusive) and from 0.0% to 25.0% (inclusive); LRs were consistently low (LR excl 0.00-0.15; LR incl 0.00-0.15). For category I (nondiagnostic), ROM surgery ranged from 16.7% to 33.3% (exclusive and inclusive), with LRs of 0.10-0.25 (exclusive) and 0.09-0.22 (inclusive) (Table 2; Fig. 2).

### Indeterminate cytology categories (TBSRTC III and IV)

**Figure 3:**
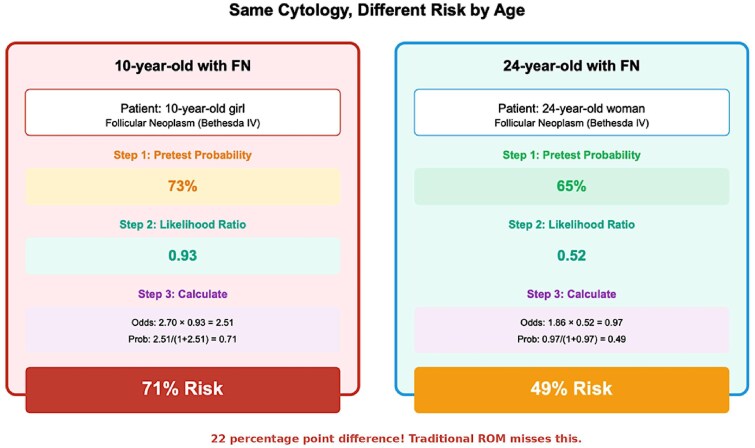
Worked clinical example illustrating the age effect on post-test risk for follicular neoplasm (Bethesda IV). Schematic example demonstrating that identical cytology can yield different absolute malignancy probabilities across age bands because the age-specific pretest probability differs. The Bayesian update used in this study is illustrated (post-test odds = pretest odds × LR, with conversion to post-test probability). Numerical inputs correspond to the age- and category-specific values in Table 2 (example shown for a 10-year-old [9-14 band] vs a 24-year-old [19-25 band]).

**Figure 4:**
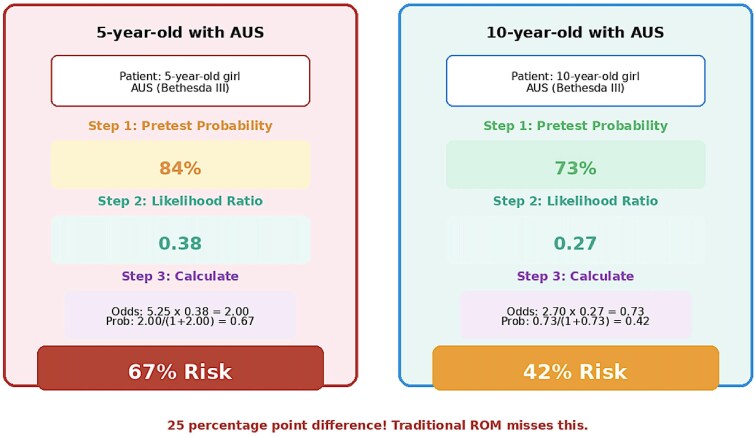
Worked clinical example illustrating the age effect on post-test risk for AUS (Bethesda III). Example calculation for AUS (III) in two pediatric age bands (0-8 vs 9-14 years), showing how differences in age-specific pretest probability and age-specific LR translate into different post-test malignancy probabilities. Numerical inputs correspond to Table 2; category counts are in Table 4. Abbreviations as in Fig. 2.

For category III (AUS), ROM surgery ranged from 42.4% to 66.7% (exclusive) and from 45.5% to 66.7% (inclusive); LRs were below 1 in all age bands (LR excl 0.27-0.41; LR incl 0.29-0.52). For category IV (follicular neoplasm), ROM surgery ranged from 48.9% to 71.4% (exclusive) and from 55.6% to 71.4% (inclusive), with LRs close to 1 (LR excl 0.52-0.93; LR incl 0.59-0.86). Accordingly, for identical category IV cytology, post-test probabilities differed materially by age (eg, 71.4% in ages 9-14 years vs 48.9% in ages 19-25 years, exclusive definition) (Fig. 3). Unlike FN, AUS post-test risk was relatively stable after age 9 (42-43%), and the higher estimate in ages 0-8 years should be interpreted cautiously due to small numbers (Fig. 4).

### Verification bias and borderline tumors (exclusive vs inclusive definitions)

Within each age band and Bethesda category, ROM surgery exceeded ROM overall. For example, in category II (benign) at ages 15-18 years, ROM overall was 5.2% versus ROM surgery of 23.4% (exclusive), and in category III (AUS) at ages 19-25 years, ROM overall was 13.5% versus ROM surgery of 42.6% (exclusive). Reclassifying borderline tumors as malignant (inclusive definition) produced small shifts in age-band pretest prevalence (maximum absolute difference 3.2% points across age bands) (Table 2).

### AUS subcategorization (nuclear atypia vs other atypia)

In the overall cohort aged 0-25 years, ROM surgery was similar for AUS with nuclear atypia (35.7% [15/42] exclusive; 42.9% [18/42] inclusive) and AUS with other atypia (36.4% [8/22] exclusive; 40.9% [9/22] inclusive) (Table 5).

**Table 5:** AUS subcategory analysis by age band (nuclear atypia vs other)

AG-G	AUS subcategory	N (All)	N (Surg.)	Malignant n (Excl)	Malignant n (Incl)	ROM% (Overall, Excl) (95% CI)	ROM% (Overall, Incl) (95% CI)	ROM% (Surg., Excl) (95% CI)	ROM% (Surg., Incl) (95% CI)
0-8	Nuclear Atypia	1	1	0	0	0.0% (0.0-97.5%)	0.0% (0.0-97.5%)	0.0% (0.0-97.5%)	0.0% (0.0-97.5%)
0-8	Other AUS	1	0	0	0	0.0% (0.0-97.5%)	0.0% (0.0-97.5%)	NA	NA
9-14	Nuclear Atypia	24	10	2	3	8.3% (1.0-27.0%)	12.5% (2.7-32.4%)	20.0% (2.5-55.6%)	30.0% (6.7-65.2%)
9-14	Other AUS	9	5	1	1	11.1% (0.3-48.2%)	11.1% (0.3-48.2%)	20.0% (0.5-71.6%)	20.0% (0.5-71.6%)
15-18	Nuclear Atypia	45	14	8	9	17.8% (8.0-31.4%)	20.0% (9.5-33.7%)	57.1% (28.9-82.3%)	64.3% (35.1-87.2%)
15-18	Other AUS	12	6	2	2	16.7% (2.1-48.4%)	16.7% (2.1-48.4%)	33.3% (4.3-77.7%)	33.3% (4.3-77.7%)
19-25	Nuclear Atypia	65	17	5	6	7.7% (2.5-17.0%)	9.2% (3.5-18.9%)	29.4% (10.3-56.0%)	35.3% (14.2-61.7%)
19-25	Other AUS	50	11	5	6	10.0% (3.3-21.8%)	12.0% (4.5-24.3%)	45.5% (16.7-76.6%)	54.5% (23.4-83.3%)
TOTAL ≤25	Nuclear Atypia	135	42	15	18	11.1% (6.4-17.7%)	13.3% (8.1-20.3%)	35.7% (21.6-52.0%)	42.9% (27.7-59.0%)
TOTAL ≤25	Other AUS	72	22	8	9	11.1% (4.9-20.7%)	12.5% (5.9-22.4%)	36.4% (17.2-59.3%)	40.9% (20.7-63.6%)

AG-G = age group (0-8, 9-14, 15-18, 19-25 years). AUS subcategory = Nuclear atypia vs Other AUS per TBSRTC (2023). N(All) = all AUS FNAs in the age-band × subcategory cell. N (Surg.) = cases in that cell that underwent surgery. M (Excl) = Number of histology-proven malignant tumors, *excluding* low-risk/borderline neoplasms, among operated cases with histology available. M (Incl) = number of malignancies + low-risk (borderline) neoplasms counted as events (in this cohort: NIFTP and tumors of uncertain malignant potential [FT-UMP/WDT-UMP]; hyalinizing trabecular tumor was prespecified but not observed). ROM% (Overall, Excl) and ROM% (Overall, Incl) = lower-bound ROM using all AUS FNAs in the cell (nonoperated counted as benign). ROM% (Surg., Excl) and ROM% (Surg., Incl) = upper-bound ROM among surgically treated cases with histology. 95% CIs for ROM use exact Clopper–Pearson. NA = not estimable; subtotals are sums within age bands. Exclusive endpoint (Excl): counts only histology-proven malignant tumors and explicitly excludes low-risk/borderline neoplasms. Inclusive endpoint (Incl): counts malignant + low-risk/borderline neoplasms as events.

## Discussion

This multi-institutional cohort of 2728 thyroid FNAs from pediatric and young adult patients provides, to our knowledge, the first age-stratified Bayesian (likelihood-ratio-based) risk framework for thyroid cytology. Four findings are central. Baseline (pretest) malignancy prevalence among surgically treated nodules decreased in a largely stepwise pattern across developmental age groups. This trend persisted in 6 of 7 leave-one-center-out analyses, with the single exception attributable to differences in referral patterns and histologic verification (Table 3), from 84.2% at ages 0-8 years to 72.8% at 9-14 years, 66.8% at 15-18 years, and 64.6% at 19-25 years under the exclusive definition (Table 2; Fig. 1). Second, the post-test risk conveyed by indeterminate Bethesda categories was age dependent, with the largest differences observed for AUS (category III) and follicular neoplasm (category IV) (Table 2; Fig. 2). Third, expressing cytology performance as age-specific LRs enables mathematically explicit conversion from age-specific pretest probability to individualized post-test probability, addressing a key limitation of single ROM tables. Fourth, verification bias was substantial: ROM estimates derived from surgical cases alone (upper-bound ROM) were consistently higher than lower-bound estimates that assume nonoperated nodules are benign, particularly for benign and indeterminate cytology (Table 2).

The age gradient in baseline risk has direct biological and clinical implications. The nearly 20%-point separation between the youngest and oldest strata (exclusive definition) indicates that age is a clinically meaningful determinant of malignancy probability before cytology is even considered (Table 2; Fig. 1). This observation is consistent with population-level data showing more invasive presentation in prepubertal children than in adolescents and young adults (4), and with pediatric molecular series showing a shift from fusion-predominant tumors at younger ages toward point-mutation-predominant tumors in older adolescents and young adults, approaching adult patterns (7-9). In practice, the implication is straightforward: the same cytology category can-and in our data does-translate into different absolute malignancy probabilities depending on age, because the baseline risk differs across age strata. For AUS (Bethesda III), post-test risk was higher in ages 0-8 years (66.7%) than in ages 9-14 years (42.4%), but the youngest estimate is imprecise because of very small numbers (*n* = 4); from ages 9-25 years, post-test risk ranged from 42.4% to 43.1% (Fig. 4).

High-risk TBSRTC categories retained strong rule-in performance across the entire age spectrum. For malignant cytology (category VI), post-test probabilities (ROM surgery) were 98.4-100.0% across age bands (exclusive definition), with very high LRs often extremely large and, in some cells, infinite because no benign surgical outcomes occurred (Table 2). For SFM (category V), post-test probabilities were 91.5-100.0% (Table 2). Although the 0-8-year stratum contained few cases, the observed ROM surgery of 100% for categories V and VI in that group suggests that frankly worrisome cytomorphology in very young children is rarely a false positive in tertiary practice (Table 2). Collectively, these results support the application of standard cytomorphologic thresholds for categories V and VI across childhood and young adulthood, with management decisions primarily driven by the diagnosis itself rather than age.

Benign cytology (category II) provided substantial rule-out information, but the magnitude of “residual risk” depended on how verification bias was handled.

Across age groups with sufficient data, benign cytology showed low LRs (LR excl 0.07-0.15), yielding post-test malignancy probabilities of 15.5-23.4% among surgically resected nodules, compared with age-specific pretest probabilities of 64.6-72.8% (Table 1). In contrast, lower-bound malignancy estimates for benign cytology across the full cohort were significantly lower (0.0-5.2%) (Table 2).

This discrepancy indicates a strong selection of clinically concerning nodules for surgery despite benign cytology. A second, nonexclusive explanation is sampling or interpretive limitation in a subset of surgically resected cases. Accordingly, benign cytology should not be interpreted based solely on a single ROM value. Instead, the true risk likely lies between the lower- and upper-bound estimates, depending on the clinical rationale for surgery (Table 2). The limited availability of ultrasound and other clinical risk features in this dataset precluded direct modeling of their contribution to this enrichment.

Indeterminate TBSRTC categories conveyed intermediate risk and showed the clearest age-related differences. For AUS (category III), upper-bound ROM (exclusive definition) ranged from 42.4% to 66.7%, highest in the youngest children, and LRs were consistently below 1 (LR excl 0.27-0.41; LR incl up to 0.52), indicating modest downshifting from the already high age-specific baseline risk (Table 2). These AUS risks exceed adult ROM ranges commonly cited for category III in the 2023 TBSRTC (6), supporting the view that AUS in children often warrants more definitive evaluation than in adults, particularly when the baseline risk is highest. For follicular neoplasm (category IV), the LRs were close to 1 (LR excl 0.52-0.93), and post-test probabilities largely tracked the age-specific pretest probability (ROM surgery (excl) 48.9-71.4%) (Table 2). This pattern highlights an important conceptual point: in pediatric practice, category IV often functions less as a “high-risk” category and more as a designation of follicular-patterned neoplasia requiring histologic assessment, with absolute malignancy probability heavily influenced by baseline risk (age stratum) rather than by the cytology result alone.

Whether AUS subcategorization adds clinically reliable risk discrimination in children remains uncertain. The 2023 TBSRTC distinguishes AUS with nuclear atypia from other atypia largely on adult evidence (6). In a pediatric-adult comparison study, malignancy among resected adult AUS nodules was higher when cytological atypia was present than when it was absent (45% [156/347] vs 23% [43/183]); in the small pediatric subset, the difference was not statistically significant (60% [12/20] vs 33% [2/6]; *P* = 0.37) (2). In a subsequent pediatric AUS subtyping study (68 nodules in 61 children), nuclear atypia was strongly associated with malignancy (59% [22/37] vs 6.5% [2/31]) (16). In our cohort, AUS subcategorization was available for 207/329 nodules (62.9%); among surgically treated AUS nodules, ROM surgery did not differ between nuclear atypia and other AUS (35.7% [15/42] vs 36.4% [8/22]) (Table 5). This discordance could reflect differences in cohort composition, interpretive thresholds or institutional practice patterns of pediatric tumors across centers. Our data do not support relying on AUS subtyping alone to stratify risk across pediatric age groups; if subtyping is reported, it should be interpreted cautiously and in context.

Our study also operationalizes recent methodological critiques of ROM tables. As emphasized by Pusztaszeri, Saieg, and Baloch (13), ROM is a post-test probability that varies with pretest prevalence and patient selection; therefore, a single ROM per category can be misleading across settings and subpopulations. The likelihood-ratio approach addresses this limitation by separating test performance (LR) from baseline prevalence and allowing explicit computation of post-test probability from a defined pretest probability (Table 2). Here, age strata provide an objective, clinically meaningful basis for pretest stratification, and the resulting age-specific LRs can be applied to generate patient-specific risks. The framework is also extensible: when additional predictors (eg, ultrasound pattern or molecular testing) are available and validated in children, LRs can be incorporated sequentially to refine post-test probability within the same Bayesian structure.

Verification bias is an inherent challenge in thyroid cytology research. In this cohort, lower-bound ROM estimates (assuming nonoperated nodules were benign) and upper-bound estimates (surgical cases only) differed by several-fold, particularly for benign and AUS cytology (Table 2). Reporting both provides transparent bounds within which the true malignancy risk is likely to reside. Although statistical corrections for verification bias (eg, Begg-Greenes methods, maximum-likelihood approaches, or multiple imputation) can be informative, they rely on assumptions about missingness and may be unstable in sparsely populated categories or those with very low false-positive rates. Future studies could apply these methods in conjunction with more standardized follow-up and, where feasible, pediatric-appropriate molecular testing to reduce reliance on surgical verification.

This study has limitations. Its retrospective, multi-institutional design introduces variability in FNA technique, cytologic interpretation, surgical thresholds, and follow-up. Center-mix sensitivity analyses (Tables 1 and 3) showed that the 0-8-year group consistently remained the highest-risk stratum, although baseline risk estimates for midadolescents shifted depending on which center was excluded, reflecting differences in referral patterns and histologic verification. Because a very small subset of FNAs (*n* = 5) were performed before local implementation of TBSRTC, their Bethesda categories were assigned retrospectively from contemporaneous descriptive reports, which may introduce a limited diagnostic misclassification. The smallest age group (0-8 years) yielded wide confidence intervals for several estimates. Clinical and imaging variables, such as biopsy indication, ultrasound features, and key patient-level risk factors, were not consistently available, limiting the ability to perform standardized multivariable modeling; follow-up for some nonoperated nodules was also incomplete. Finally, indications for surgery and surgical thresholds varied across centers and time, contributing to differential histologic verification. To account for this verification bias, we report both lower- and upper-bound ROM estimates. Strengths include the large international cohort spanning four developmental age groups, consistent use of 2023 TBSRTC categories, explicit bracketing of verification bias using lower- and upper-bound ROM estimates, and provision of age-specific LRs that translate cytology results into patient-specific post-test risks.

In conclusion, age is a major determinant of both baseline and post-test malignancy risk in pediatric and young adult thyroid cytology. An age-stratified LR framework enables quantitative and transparent risk adjustment, moving beyond reliance on single ROM values. This approach provides a rigorous and portable foundation for individualized clinical decision-making and for the development of future multimodal pediatric thyroid nodule risk models that integrate standardized imaging, clinical, and molecular data.

## Data Availability

De-identified data that support the findings of this study are available from the corresponding author upon reasonable request and subject to institutional approvals.
